# Production of Exopolysaccharides and İndole Acetic Acid (IAA) by Rhizobacteria and Their Potential against Drought Stress in Upland Rice

**DOI:** 10.4014/jmb.2401.01035

**Published:** 2024-05-14

**Authors:** Tetty Marta Linda, Jusinta Aliska, Nita Feronika, Ineiga Melisa, Erwina Juliantari

**Affiliations:** Department of Biology, Faculty of Mathematics and Natural Sciences, Riau University. Kampus Bina Widya Km. 12, 5 Simpang Baru Pekanbaru, Riau Province 28293, Indonesia

**Keywords:** Exopolysaccharide, indole acetic acid, *Klebsiella variicola*, peat land, rhizosphere bacteria, upland rice

## Abstract

Peatlands are marginal agricultural lands due to highly acidic soil conditions and poor drainage systems. Drought stress is a big problem in peatlands as it can affect plants through poor root development, so technological innovations are needed to increase the productivity and sustainability of upland rice on peatlands. Rhizobacteria can overcome the effects of drought stress by altering root morphology, regulating stress-responsive genes, and producing exopolysaccharides and indole acetic acid (IAA). This study aimed to determine the ability of rhizobacteria in upland rice to produce exopolysaccharides and IAA, identify potential isolates using molecular markers, and prove the effect of rhizobacteria on viability and vigor index in upland rice. Rhizobacterial isolates were grown on yeast extract mannitol broth (YEMB) medium for exopolysaccharides production testing and Nutrient Broth (NB)+L-tryptophan medium for IAA production testing. The selected isolates identify using sequence 16S rRNA. The variables observed in testing the effect of rhizobacteria were germination ability, vigour index, and growth uniformity. EPS-1 isolate is the best production of exopolysaccharides (41.6 mg/ml) and IAA (60.83 ppm). The isolate EPS-1 was identified as *Klebsiella variicola* using 16S rRNA sequencing and phylogenetic analysis. The isolate EPS-1 can increase the viability and vigor of upland rice seeds. *K. variicola* is more adaptive and has several functional properties that can be developed as a potential bioagent or biofertilizer to improve soil nutrition, moisture and enhance plant growth. The use of rhizobacteria can reduce dependence on the use of synthetic materials with sustainable agriculture.

## Introduction

Peatlands in Indonesia are widely utilized for agricultural development, such as the cultivation of upland rice (*Oryza sativa* L.) to achieve self-sufficiency in staple foods. Although peatlands are considered marginal lands and prone to degradation, they can be productive agricultural lands if managed properly [1]. Peatland management has many obstacles, including the high content of organic material, acid pH, low base saturation, and high solubility of Al, Fe, and Mn, which results in the unavailability of macro-nutrients in sufficient quantities and an increase in micro-nutrients that can be toxic to plants [2]. In addition, the low content weight of peat can cause peat to lose its ability to absorb water (irreversible drying) and become a dry organic matter that is not suitable for use as a growing medium [3].

In terms of upland rice, a major challenge for farmers in growing rice on peatlands is the availability of water for plant growth. In addition, the low content weight of peat can cause peat to lose its ability to absorb water (irreversible drying) and become a dry organic matter that is not suitable for use as a growing medium [3]. In the dry season, there will be drought, which is not ideal for growing rice, so technological innovations are needed to increase the productivity and sustainability of upland rice on peatlands. Most plants interact directly with various microbes to improve their drought tolerance and survival [4]. Upland rice is a rice ecotype that differs from irrigated rice ecotypes. It is adapted to drought stress conditions [5].

To obtain microbes that can survive in peatland, it is necessary to isolate microbes from marginal lands because these microbes are more adaptive and have a number of functional characteristics that can increase the availability of nutrients for plants. One of the efforts that can be made as an alternative to overcome these problems is the provision of rhizosphere bacteria or plant growth-promoting rhizobacteria (PGPR) that produce extracellular polysaccharides or exopolysaccharides and indole acetic acid (IAA) phytohormones [6-8].

Exopolysaccharides is a mix of different metabolites exudated by the bacteria released in response to physiological stress in the environment consisting of carbohydrates, proteins, and lipids [9]. Exopolysaccharides produced by rhizobacteria from seven upland rice exposed to salt in Kedah, Malaysia, can help bind Na+ in the soil to reduce plant exposure to ions in saline conditions, produce IAA, bind nitrogen, dissolve phosphate and potassium, increase the rate of photosynthesis, and increase nutrient uptake [10]. The effect of exopolysaccharides-producing *Bacillus* strains from *Cistanthe longiscapa* from the Atacama Desert, Chile applied to tomato seeds can restore drought and increase seed height and weight gain [11]. *Bacillus velezensis*, which produces exopolysaccharides and has 1-aminocyclopropane-1-carboxylic acid (ACC) deaminase activity from the rhizosphere of corn, could increase the rate of photosynthesis, water use efficiency, stomatal conductance, increased transpiration, and corn roots colonization [12].

Numerous studies have reported about bacterial species produce exopolysaccharides, such as *Proteus penneri*, *Pseudomonas aeruginosa*, and *Alcaligenes faecalis*, that can increase corn growth by improving soil moisture contents. The exopolysaccharides hold the water in the soil surrounding the plant roots and soil dries more slowly, also protect the bacteria from desiccation and fluctuations in water potential [13]. *Azotobacter chroococcum* can increase plant height, dry root weight, and N uptake in soybean [14]. *Klebsiella* sp. LW-13, *Klebsiella pneumoniae* strain DSM 30104, and *Bukholderia anthina* strain MYSP113 can increase the aggregate stability of sandy soil [15]. In addition, *Enterobacter* sp. RZS5 has a tolerance to high concentrations of heavy metals such as Mn^2+^, Ni^2+^, Zn^2+^, Cu^2+^, CO_2_+, dan Fe^2+^ that evidenced by an increase in promoted seed germination, shoot height, root length, number of leaves and chlorophyll content of *Triticum aestivum* and *Arachis hypogaea* seed [16]. Some EPS-producing bacteria are also proven as potential mercury bioaccumulators [17], antibacterials [18], phytostabilization of metal-contaminated soil [19] and production of the phytohormone IAA.

IAA is the most common plant hormone of the auxin class and regulates various plant growth processes. IAA hormone produced by bacteria can increase the plant's root hairs and lateral roots. Nutrient absorption in the soil becomes maximized so that plants will grow faster [20]. It has been reported that 34 isolates of exopolysaccharides-producing bacteria from the rhizosphere of potato (*Solanum tuberosum* L.) in Nutrient Broth (NB) + L-tryptophan medium for three days at 28°C produced IAA of 0.4-21.14 mg/l [21].

Various studies report that rhizosphere bacteria that can produce IAA are very diverse. Identification of the species of bacteria can be determined using the 16S rRNA gene [22]. It has been reported that research used 16S rRNA marker to identify bacteria from the rhizosphere of *Lotus corniculatus* from grasslands into the genus *Mesorhizobium* and *Bradyrhizobium* with 99% homology [23]. Research identified bacteria associated with Origanum vulgare roots using the same primers (8F and 1492R) classified into the phylum α-Proteobacteria, γ-Proteobacteria, Firmicutes and Actinobacteria with 97-100% homology [24].

In this study, eight the bacterial isolates used came from microbiology and bioprocessing laboratory collection, Riau University. The bacterial isolates were collected from the rhizosphere of upland rice from the peat soil of Sei Cingam Village, Rupat District. The eight bacterial isolates obtained have Gram-negative characteristics, with slimy/mucoid colony surfaces. Based on previous studies, we hypothesize that rhizosphere bacteria can produce exopolysaccharides and IAA can increase nutrient availability to upland rice growth. There is still limited information about rhizosphere bacteria in peatlands and their potential. This study aims to determine the ability of bacterial isolates to produce exopolysaccharides and IAA. Furthermore, the selected isolates were identified molecularly based on the 16S rRNA gene (ribosomal ribonucleic acid) gene. The management of upland rice cultivation on peatlands will provide empirical information to local farmers that can be used as a source of inspiration to enrich the rice management system on peatlands.

## Materials and Methods

### Preparation and Culture Rhizosphere Bacteria

The bacterial isolates used came from the long-term storage collection of the microbial gene bank of the Microbiology Laboratory, Department of Biology, Faculty of Mathematics and Natural Sciences, Riau University. A total of 0.1 ml of rhizosphere bacterial isolate was mixed with 9 ml of Nutrient Broth (NB) media and then incubated for 24 h on a shaker incubator at 30°C speed at 120 rpm. A total of 1 ml of the isolate was taken and poured into a petri dish, then added with 15 ml of Nutrient Agar (NA) media. Incubation was carried out for 24 h at room temperature. Bacterial colonies were rejuvenated with the streak quadrat technique and then grown on slant agar to be stored at 4ºC for the next stage of work.

### Biochemical and Physiological Characterization of Bacteria

Physiological characteristics include carbohydrate fermentation tests using glucose, fructose, and sucrose. The carbohydrate medium with a composition of 2 grams; 2 grams of tryptone; 1 gram of NaCl; 0.0036 grams of red phenol indicator in a test tube containing Durham tube. The Carbohydrate medium was inoculated with bacteria and incubated for 24-48 h. Changes in medium color to yellow and gas formation indicate positive fermentation in producing acid. Each bacterial cell was then subjected to Gram staining and catalase test. The bubbles on the isolate on the glass object indicate a positive result of producing the enzyme catalase.

### Exopolysaccharide (EPS) Production of Rhizosphere Bacterial

Each bacterial inoculum was grown into yeast extract mannitol broth (YEMB) medium. A total of 5 ml of each treatment with a bacterial population of 10^8^ CFU/ml was inoculated into an Erlenmeyer containing 50 ml of YEMB medium and incubated at 28°C in a shaker incubator at 120 rpm for incubation three days. At the end of incubation, 1 ml of each medium was added with 500 μl EDTA, then centrifuged at 9,000 ×*g* for 10 min. The supernatant was added with acetone in the ratio of 1:3, then homogenized and centrifuged again at 13,000 ×*g* three times 20 min. The exopolysaccharide at the bottom of the tube was poured into Whatman paper. The formula for calculating the exopolysaccharides is the weight of filter paper (with exopolysaccharides) minus the weight of filter paper (without exopolysaccharides) [16, 25, 26].

### Fourier Transform Infra-Red (FTIR)

The pellet of exopolysaccharides produced by EPS-1 were characterized for their molecular structure using FTIR spectroscopy (IRPrestige-21 Shimadzu). The exopolysaccharide was mixed with potassium bromide (KBr). FTIR examined the structure exopolysaccharides from isolate EPS-1 at spectra of 4,000 to 500 cm^-1^. Then, the spectra were recorded using IR software.

### Qualitative and Quantitative Test of Indole Acetic Acid (IAA) Production In Vitro

A total of 1 ml of each bacterial inoculum (10^8^ CFU/ml) was put into 4 ml of NB medium + 500 μg/ml L-tryptophan [26, 27] and incubated for three days on a shaker incubator at 150 rpm. After incubation, culture then centrifuged at 3,000 ×*g* for 30 minutes. A total of 1 ml of supernatant was transferred into a sterile test tube, and 4 ml of Salkowski reagent was added, then incubated in the dark for 30 min. A qualitative test was done by observing the color change of the solution to pink. While the quantitative test was done by measuring the absorbance value using a spectrophotometer at a wavelength of 530 nm compared with the IAA standard curve. Synthetic IAA was made serially, where 5 mg was dissolved into 50 ml of ethanol to obtain a concentration of 70 ppm and added methanol to 1,000 μl, then added Salkowski reagent as much as 4 ml. The solution was then homogenized and incubated in the dark for 60 min. The absorbance value of spectrophotometry is made a standard curve of the IAA solution.

### Molecular Identification of Rhizosphere Bacterial

Rhizosphere bacteria were identified using 16S rRNA gene. DNA was extracted by using a PrestoTM Mini gDNA Bacteria Kit. DNA was amplified by using 16S rRNA universal primers: 8F (5'-AGA GTT TGA TCC TGG CTC AG-3') and 1492R (5'-GGT TAC CTT GTT ACG ACT T-3'). The PCR reaction consisted of an activation step of pre-denaturation 94°C for 2 min followed by 35 cycles of denaturation 94°C for 30 sec, an annealing step of 49.85°C for 1.30 m, and an extension step of 72°C for 1 m. The amplified products were then purified by PCR Clean-Up or Gel Extraction depending on visualization results for Single Pass DNA Sequencing.

### Rice seed Germination Test with Rhizosphere Bacterial

The rice seed germination test uses the method of [8] with modifications used 5 repetitions. Sterile rice seeds were soaked in 50 ml of rhizobacterial inoculum (population 10^8^ CFU/ml) for 24 h and distilled water for 24 h as a control. Then 20 seeds were germinated in a container filled with moist filter paper. Measurements were taken on the 7 days after sowing. The parameters observed were the viability and vigour of upland rice seeds.

### Data Analysis

Data from exopolysaccharide and IAA production was analyzed using and descriptively presented in tables and figures. The germination test analysis using SPSS program version 25. Meanwhile, sequence analysis was performed with the BLASTn program to determine the percentage of similarity of isolates to reference isolates in GeneBank. The alignment results were analyzed using the Basic Local Alignment Search Tool (BLAST) program through the NCBI GeneBank site and obtained the identity of bacterial isolates. Sequencing results would be aligned with Clustal W program which was inside MEGA 11 and then the cladogram would be built based on the alignment of sequence data. Phylogenetic tree construction using the Unweighted Pair Group with Arithmetic Mean (UPGMA) was carried out with the MEGA 11 program with the bootstap 1000x.

## Results

### Bacteria Characterization

**Fig. 1:**
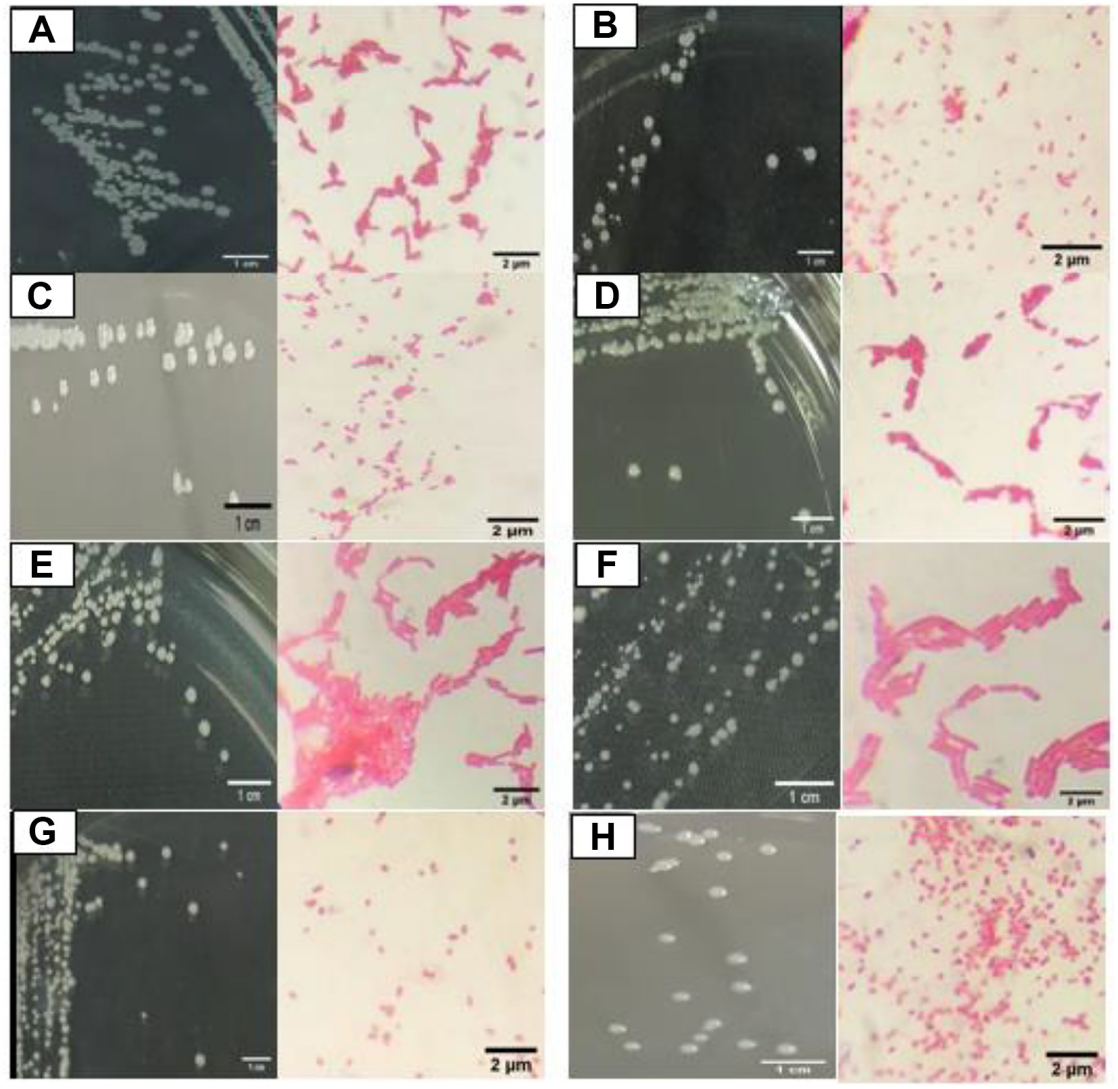
Colony and cell morphology of rhizosphere bacterial isolates of upland rice on NA media for 24 h incubation. (**A**) EPS-1. (**B**) EPS-2. (**C**) EPS-3 (**D**) EPS-4 (**E**) EPS-5. (**F**) EPS-6. (**G**) EPS-7. (**H**) EPS-8.

The cell shapes observed varied between bacilli and cocci. Biochemical and physiological characteristics showed positive results in all isolates in the catalase and carbohydrate fermentation tests (Table 1). The eight isolates of upland rice rhizosphere bacteria showed the character of Gram-negative bacteria as evidenced by the mucoid produced in the 3% KOH test. The mucoid is formed due to the rupture of bacterial cell walls in a highly alkaline solution [29]. The results obtained cell forms of bacilli (EPS-1, EPS-3, EPS-5, and EPS-6) and cocci (EPS-2, EPS-7, and EPS-8) from eight isolates of rhizosphere bacteria. All isolates are classified as Gram-negative with characteristic pink cells (Fig. 1).

### Exopolysaccharide Production of Rhizosphere Bacterial

EPS-1 isolates were able to grow well in the YEMB medium. The highest exopolysaccharide yield by isolate EPS-1 was 41.6 mg/ml (Table 2). The use of YEMB medium in this study was carried out because some bacteria around the plant can use carbon and nitrogen sources in the medium to produce exopolysaccharides. YEMB medium contains a carbon source from mannitol and a nitrogen source from yeast extract. Yeast extract is the best nitrogen source for exopolysaccharide production due to its high amino acids for bacterial growth [30]. Exopolysaccharides produced by bacteria can be influenced by the species of isolate, type of medium, pH, temperature, and incubation time [31].

### FTIR

**Fig. 2:**
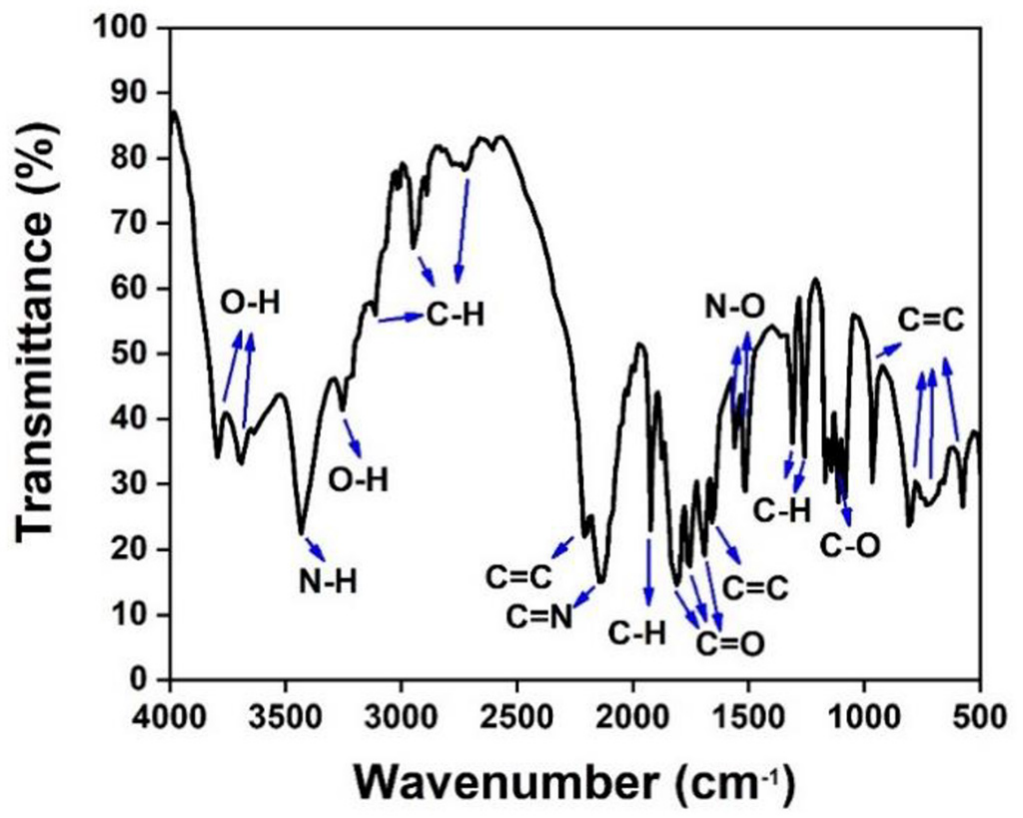
Fourier transform infrared spectroscopy (FTIR) analysis of crude extract strains EPS-1 of endophytic bacteria.

FTIR spectrum from pellet of exopolysaccharides EPS-1 bacterial isolate showed various functional groups. Hydroxyl groups (O-H) are present at wavelengths 3793.85, 3692.19, and 3255.72 cm^-1^, and N-H groups as amine groups are present at wavelengths 3431.22 cm^-1^. The wavelengths of 3111.60, 2724.38, 1924.16, 1312.03, and 1257.58 cm^-1^ were expressed as C-H groups. The wavelengths of 2209.28, 1652.75, 962.65, and 577.65 cm^-1^ contained C=C groups. The C=N functional group is present at a wavelength of 2135.48 cm^-1^, and the C=O group is at 1811.51 and 1691.57 cm^-1^. N-O groups are found at wavelengths of 1561.41 and 1515.59 cm^-1^. In addition, C-O groups are present at wavelengths of 1169.84 and 1084.01 cm^-1^ (Fig. 2).

### Indole Acetic Acid (IAA) Production by Rhizosphere Bacteria of Upland Rice Qualitatively

The analysis of IAA hormone production results based on the test color indicator obtained four IAA-producing isolates that change color when reacted with the Salkowski reagent. Two isolates produced pink color (EPS-1 and EPS-6), two isolates had light pink color (EPS-4 and EPS-7), and four other isolates (EPS-2, EPS-3, EPS-5, and EPS-8) did not change color (Table 2). The color change indicates the formation of IAA by rhizosphere bacteria. The color change reaction in isolates EPS-1, EPS-6 and EPS-4, and EPS-7 indicates the ability of these bacterial isolates to metabolize L-tryptophan into IAA with the enzymes tryptophan monooxygenase, IAM hydrolase, indole-pyruvate decarboxylase and IAAI dehydrogenase [32].

Some other studies that use the Salkowski reagent to detect IAA production, such as bacterial isolates from the rhizosphere of *Syzygium aromaticum* L. on Tryptic Soy Agar (TSA) media after incubation for 30 min produced eight pink isolates [33]. Three isolates isolated from rice plants on NA media with the addition of tryptophan after incubating for 30 min produced a pink color [34].

IAA-producing bacteria will be red due to the reaction between IAA and with Salkowski reagent, namely Fe, to form a complex compound [Fe_2_(OH)_2_(IA)_4_] [33]. The more intense the pink color formed, the higher the concentration of IAA produced [35]. The combination of Fe and H_2_SO_4_ (sulfuric acid) contained in the Salkowski reagent is a single reagent that can spur sensitivity in determining the formation of IAA.

The IAA concentration of each bacterium was measured based on the standard curve. The purpose of measuring the standard curve is to obtain an equation to calculate the concentration of IAA in the supernatant. Based on spectrophotometric measurements, the IAA standard solution curve shows the relationship between the IAA standard solution (x) and the level of absorption (y) with the regression equation y = 0.0165x + 00444. The results of measuring the concentration of IAA using NB + L-tryptophan medium for each rhizosphere bacteria known for only three isolates that produce IAA: isolates EPS-1, EPS-6, and EPS-7. Isolate EPS-1 is the highest isolate with IAA, while isolate EPS-6 is the lowest isolate that produces IAA. EPS-1 isolate the highest IAA producer is 60.83 ppm (Table 3).

### 16S rRNA Gene Sequence Rhizosphere Bacteria

**Fig. 3:**
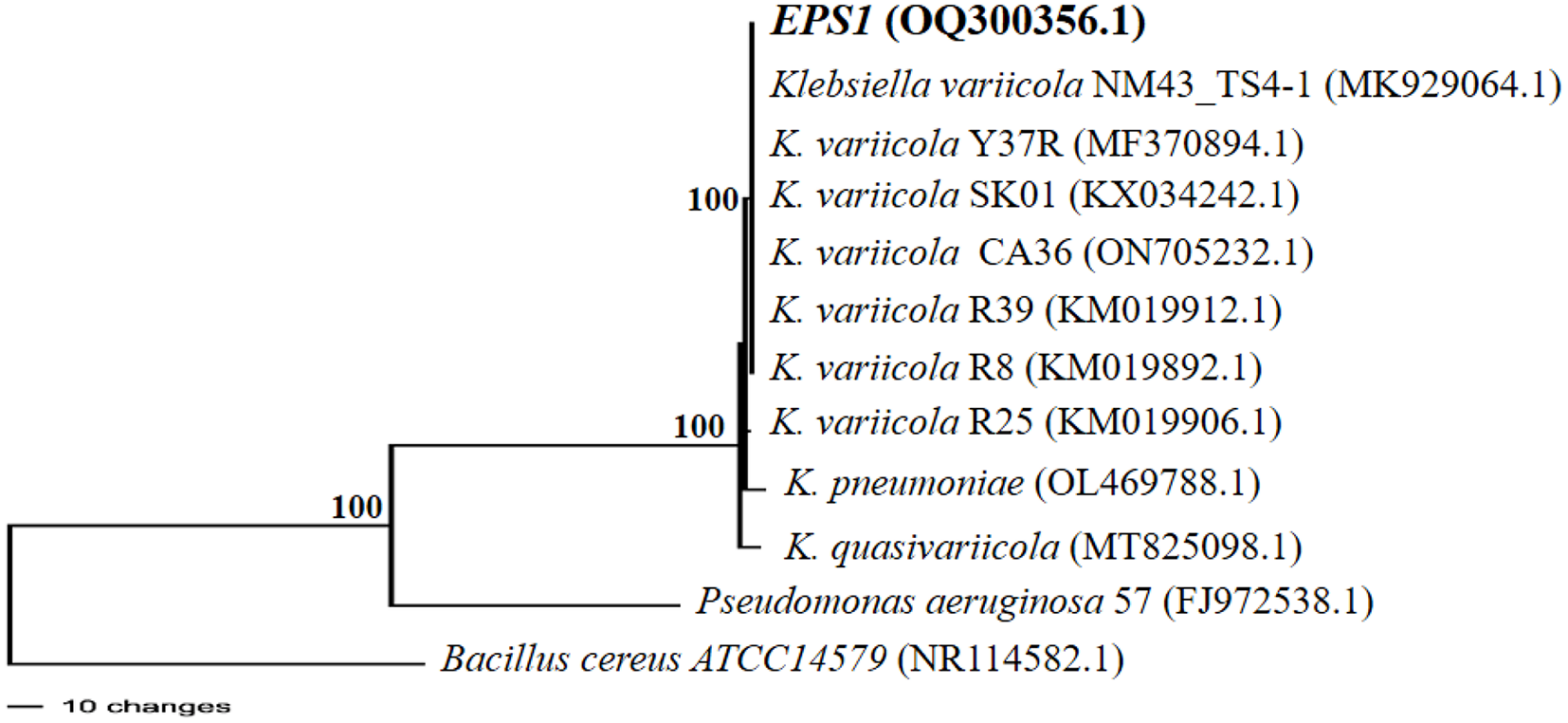
Phylogenetic tree of bacterial isolates EPS-1 based on distance matrix and UPGMA method with bootstrap 1000 replications.

Bacterial isolates (EPS-1) with the highest ability to produce exopolysaccharide and IAA were identified using the 16S rRNA gene [36]. Based on the analyzed using the Basic Local Alignment Search Tool (BLAST) program, EPS-1 bacterial isolate has similarity (100%) with *Klebsiella variicola* (Fig. 3). Based on [37], bacteria from the rhizosphere of sugarcane obtained *Klebsiella* bacterial genus with Gram-negative characteristics in the form of rods that have no color/pigment, small round colonies with a high elevation and smooth surface, can dissolve phosphate, produce Indole Acetic Acid (IAA) and potentially encourage germination/PGPR.

### Rice Seed Germination Test with Rhizobacterial Inoculum

In this study, rice seeds viability (germination) and vigor (vigor index and growth uniformity) were observed. The germination test results of field rice seeds treated with soaking inoculum of rhizobacterial isolate EPS-1, identified as *Klebsiella variicola*. The test results gave an effect that was significantly different from the control on the ability of germination, vigor index, and uniformity of growth (Table 4).

Soaking rhizobacteria for 48 h in Konawe and Inpari 10 varieties of upland rice seeds after incubation for seven days obtained the highest germination, vigor index, and uniformity of growth by *Serratia* CMN175 and *Bacillus* CKD061 [38]. In addition, soaking the endo-rhizobacterial consortium isolate Be02 + isolate PKLK5 + *Bacillus* sp. CKD061 for ± 24 h to local upland rice seeds of Momea cultivar after incubation for seven days resulted in the highest germination rate of 85.33% [28]. Different rice varieties, bacterial isolates and incubation time for seed soaking affect the amount of germination.

## Discussion

A good physical environment is closely related to the flow of nutrients and water into plant roots, aeration and soil porosity favourable for plant growth. Stable soil aggregates will create a good physical environment for plant growth [39]. However, in peat soil during the dry season, the land will dry out, making it very difficult for plants to adapt. Exopolysaccharides play an important role in forming stable soil aggregates because exopolysaccharides can increase water retention and have properties as a gelling agent [40].

Exopolysaccharides protects plants from drought stress by maintaining plant-microbe interactions [41]. In this study, EPS-1 is the best isolate from upland rice roots that produces the highest exopolysaccharides, so this isolate can be the best solution for drought-stressed agriculture in marginal lands such as peatlands. Rhizobacteria can overcome the drastic effects of water stress by increasing exopolysaccharides production and forming rhizosheaths around the roots, thus protecting against dehydration [41]. The application of exopolysaccharides-producing rhizobacteria is proven to help alleviate water shortage, thereby improving global food security [42].

The variety of ingredients in EPS contributes special qualities like improved water absorption and increased bacterial retention in the soil through aggregate formation [43]. Apart from soil aggregation, exopolysaccharide (EPS) production also helps in increasing water permeability, nutrient uptake by roots, soil stability, and soil fertility during drought stress [44]. The amount of exopolysaccharide produced depends on the species of bacteria, the source of nutrients and the environmental conditions of growth. Nutrients in the medium are helpful as cell formation and energy sources for bacteria to produce exopolysaccharides.

The principle of FTIR is bonding groups vibrate at characteristic frequencies that can be used to detect functional groups and characterize covalent bonds [21]. The FTIR results of bacterial EPS-1 show that the components of exopolysaccharides generally consist of a class of polysaccharides, sugars, and proteins.

The water-loving nature of exopolysaccharides is associated with the presence of hydroxyl content in bacteria [45, 46]. N-H groups as amines are one part of the exopolysaccharides protein structure. The presence of hydroxyl and amine groups in the composition of exopolysaccharides compounds also plays a role in overcoming heavy metal remediation in soil or protecting bacteria from heavy metal contamination [47]. C-H groups are groups of methyl groups found in hexose (glucose and galactose) or deoxyhexose (rhamnose and fructose) [48, 49]. The C=C group is an alkyne group as a carbon source provider that plays a role in heavy metal remediation [47, 50]. The C-N group is thought to be a form of amine that has an important role in binding metal ions to intact cells and exopolysaccharides-free cells [51]. The C=O group is a carboxylic acid group constituent of exopolysaccharides proteins, carboxylic acids in exopolysaccharides also play a role in ensuring affinity for oppositely charged molecules such as heavy metals [10, 46]. The N-O group is a nitro compound. The C-O group is a form of ether constituent of polysaccharides [52]. Polysaccharide components are generally found as sugar subunits, such as glucose, mannose, and galactose [53].

Further study in this research is the potential of rhizobacteria in producing ındole acetıc acıd (IAA). IAA production testing was carried out using Nutrient Broth media with the addition of L-tryptophan. The highest IAA concentration was found in EPS-1 as well. The results of IAA obtained were higher than the study [54], which examined the ability of IAA-producing bacterial isolates from peat soil in Kalimantan, Indonesia and [55] which examined the ability of IAA-producing bacterial isolates from peat soil of oil palm land in north Sumatra, Indonesia.

Growth media affects the production of IAA produced [56]. This is in line with the statement of [57] that adding L-tryptophan into bacterial growth media can increase the biosynthesis of IAA by bacteria.

Rhizobacteria in the root area can synthesize secondary metabolites of IAA because this area contains a lot of root exudates containing tryptophan, where tryptophan can come from root exudates or damaged cells [58]. Incubation time can also affect the concentration of IAA produced by bacteria. The production of IAA by bacterial isolates from soybean root soil samples on Tryptic Soy Broth (TSB) media with the addition of 0.5 ml L-tryptophan with incubation times of 0, 24, 48, and 72 h resulted in the highest concentration of IAA in found at 48-72 h incubation time, and significantly different from the incubation time of 24 h [33]. It is suspected that the time range is a stationary phase. IAA production will increase when growth conditions decrease, limited carbon availability, and in an acidic pH environment. These conditions occur when bacteria enter the stationary phase [59]. IAA produced by bacterial isolates from the rhizosphere of Thai jasmine rice plants on NB + L-tryptophan media with an incubation time of 48 h, producing IAA 37.92-46.97 ppm. Based on this explanation, it can conclude that the type of isolate, production media, the concentration of tryptophan addition, and incubation time affect the amount of IAA produced [60].

In supporting plant growth, rhizobacteria can simultaneously produce exopolysaccharides and IAA. Exopolysaccharides produced by PGPR will form a biofilm that will connect PGPR with the root appendix to take up some important nutrients so that they can be utilized by plants and prevent root damage by pathogens [61]. Exopolysaccharides have properties as soil aggregate stabilizers that create a good physical environment for plants and are related to the flow of nutrients and water into the roots, aeration, and soil porosity to support plant growth [62]. Meanwhile, IAA produced by rhizosphere bacteria will increase the length and surface of the roots so that plants will get better access to nutrients in the soil [22]. The interaction between these two compounds can be maximally utilized to support plant growth. The isolate EPS-1 was identified as *Klebsiella variicola* strain NM4_TS4-1 using 16S rRNA gene sequencing and phylogenetic analysis with 100% homology. These identification results are also supported by identification based on biochemical and physiological characteristics. Isolate EPS-1 is classified as Gram negative with characteristic pink cells as in the *Klebsiella* genus. *Klebsiella* sp. D5A genome has genes that contribute to plant growth-promoting (PGP) traits such as indole-3-acetic acid (IAA) biosynthesis [63]. The most effective rhizospheric bacterium for creating IAA was found to be the strain of *Klebsiella pneumonia*, which is stimulated to produce IAA by L-tryptophan [64]. Other than that IAA produced by *Klebsiella variicola* AY-13 has a significant effect on the capability of this bacterium to stimulate growth of primary roots and induced adventitious roots in soybean [7].

Rhizobacteria play an important role in improving the rhizosphere microenvironment of upland rice seedlings under drought and acid stress. Drought, especially in peatlands, affects plant water potential and turgor, disrupting plant function and altering physiological and morphological traits [65]. *Klebsiella variicola* (EPS-1) isolates have been shown to improve the growth of upland rice seeds from peatlands with exopolysaccharides and IAA production. Plant drought stress tolerance mechanism provided by bacteria is the accumulation of soluble sugars. Exopolysaccharides, a sugar polymer secreted by the microbes out of the cell, is a main function for bacteria as cell protection from drought stress [66]. And as phytohormones play a crucial role in plant growth and development, IAA can help plants cope with drought stresses [67, 68].

Rhizobacteria can produce the hormone IAA, which spurs the growth of rice plant roots [69]. Rhizobacteria can increase the viability and vigor of upland rice seeds with the production of IAA, cytokinins and gibberellins and suppress fungi and other bacteria with antibiosis, thus acting as a growth promoter [38]. In addition, endo-rhizobacteria can produce growth hormones in the form of IAA, resulting in an increase in the root length of local upland rice seeds [28].

From this study, it is discovered that *K. variicola* performs various biological functions to enhance the growth and germination of upland rice seeds from dry peatlands, namely by producing exopolysaccharides and IAA. No previous studies have provided this information. Based on research [70], *K. variicola* was able to cope with drought-stressed plants by accumulating glycine betaine (GB) and choline involved in osmotic adjustment and protection of essential biomolecules. *K. variicola* can improve the rhizosphere soil microenvironment of maize seedlings and consequently enhance maize seedling growth, especially under saline-alkali stress conditions [71].

From this study, *K. variicola* was shown to be more adaptive and have some functional properties that can be developed as a potential biological agent or biofertilizer to improve soil nutrition, moisture, and enhance plant growth. This research contributes significantly to developing microbial fertilizers suitable for marginal lands with acidic and drought-prone soils and can reduce dependence on the use of synthetic materials with sustainable agriculture.

## Figures and Tables

**Table 1:** Biochemical and physiological characteristics of EPS-producing Gram-negative bacteria.

Isolate Code	Cell Shape	Catalase Test	Carbohydrate Fermentation		
			Glucose	Fructose	Sucrose
EPS-1	Bacili	+	+	+	+
EPS-2	Coccus	+	+	+	+
EPS-3	Bacili	+	+	+	+
EPS-4	Bacili	+	+	+	+
EPS-5	Bacili	+	+	+	+
EPS-6	Bacili	+	+	+	+
EPS-7	Coccus	+	+	+	+
EPS-8	Coccus	+	+	+	+

**Table 2:** EPS concentration measurement results of rhizosphere bacterial of upland rice grown on NB+Triptofan medium with 72 h incubation time.

Isolate Code	EPS production (mg)
EPS-1	41.60 ± 0.51
EPS-2	4.33 ± 0.17
EPS-3	6.22 ± 0.49
EPS-4	18.86 ± 0.53
EPS-5	30.53 ± 0.32
EPS-6	7.00 ± 0.59
EPS-7	14.90 ± 0.91
EPS-8	14.16 ± 0.32

**Table 3:** Measurement Results of IAA Concentration of Rhizosphere Bacterial Isolates upland Rice grown on medium NB + Tryptophan incubation time of 72 h.

Isolate Code	Color	IAA Production (ppm)
EPS-1	Pink	60,83 ± 4.65
EPS-2	No discoloration	-
EPS-3	No discoloration	-
EPS-4	Light pink	1.58 ± 0.35
EPS-5	No discoloration	-
EPS-6	Pink	49.95 ± 3.22
EPS-7	Light pink	7.03 ± 1.99
EPS-8	No discoloration	-

**Table 4:** Average percentage of viability and vigor of rice seeds immersion by rhizosphere bacteria.

No	Treatment	Germination ability (%)	Vigor index (%)	Growth uniformity (%)
1	Control	56,67 ± 7,64^a^	56,67 ± 7,64^a^	56,67 ± 7,64^a^
2	EPS-1 (*Klebsiella variicola*)	90,00 ± 5,00^b^	90,00 ± 5,00^b^	90,00 ± 5,00^b^

Numbers followed by the same letter in the same column indicate not significantly different in the DMRT further test at the 5% level.
